# Functional Analysis of Adipokinetic Hormone and Its Receptor Genes in Regulating Energy Metabolism Under Stress Conditions in *Dendroctonus armandi*

**DOI:** 10.3390/ijms27062724

**Published:** 2026-03-17

**Authors:** Linjun Wang, Ming Tang, Hui Chen

**Affiliations:** 1 College of Forestry, Northwest A&F University, Yangling 712100, China; 2020060253@nwafu.edu.cn; 2 State Key Laboratory for Conservation and Utilization of Subtropical Agro-Bioresources, College of Forestry and Landscape Architecture, South China Agricultural University, Guangzhou 510642, China

**Keywords:** Chinese white pine beetle, starvation and temperature stresses, adipokinetic hormone (AKH), adipokinetic hormone receptor (AKHR), RNAi

## Abstract

*Dendroctonus armandi* is a major primary pest of Chinese white pine in the Qinling–Bashan forest region. By feeding on the phloem and vectoring symbiotic fungi that cause blue stain in the sapwood, it drives rapid decline and mortality of host trees. As a key wood-boring forest insect, its outbreaks are closely linked to adaptive strategies in energy metabolism. Adipokinetic hormone (AKH) is a highly conserved insect neuropeptide and plays a major role in regulating energy metabolism. This study aimed to determine how the *AKH* gene regulates energy use in *D. armandi* under different stress conditions. We cloned the *DaAKH* gene and its receptor gene, *DaAKHR*, from *D. armandi*. *DaAKH* and *DaAKHR* showed the highest expression in emerged adults and the lowest levels in pupae. In larvae and in adult males and females, *DaAKH* transcripts were predominantly expressed in the head, whereas *DaAKHR* was enriched in the fat body. Under starvation and cold stress, *DaAKH* and *DaAKHR* expression were significantly upregulated; under heat stress, expression first increased and then decreased. Across stress treatments, RNAi significantly downregulated *DaAKH* and *DaAKHR* expression in *D. armandi*. Under starvation, RNAi reduced mortality, lowered lipid metabolism, and led to lipid accumulation, thereby mitigating premature energy depletion and starvation-induced death. By contrast, under heat and cold stress, RNAi significantly increased mortality, significantly reduced triglyceride and glycogen consumption, and suppressed metabolism. These results indicate that *DaAKH* and *DaAKHR* regulate energy allocation under starvation stress and help maintain adaptive capacity under temperature stress in *D. armandi*. By tuning energy metabolism, *DaAKH* and *DaAKHR* help resist environmental stress and maintain reproduction and population size. This study advances understanding of the physiological responses and molecular mechanisms of *D. armandi* under stress conditions and provides a new avenue for metabolism-targeted control.

## 1. Introduction

Energy allocation and metabolic coordination are among the key capacities that enable insects to withstand environmental stressors [1,2]. Starvation alters insect survival strategies and physiological adaptation by affecting energy reserves and allocation, metabolic pathways, and hormonal regulatory networks [3]. In *Tribolium castaneum*, metabolic rate declined after 72 h of prolonged starvation [4]. In *Drosophila*, insulin-like peptides (ILPs) are key neuropeptides that regulate the starvation response, and their secretion is suppressed under nutrient deprivation [5]. In *Bombyx mori*, starvation activates the FoxO transcription factor, triggering alterations in both the insulin signaling pathway and the MAPK cascade [6]. The Neuropeptide F/Neuropeptide F receptor (NPF/NPFR) and short neuropeptide F/short neuropeptide F receptor (sNPF/sNPFR) signaling pathways also play critical roles in orchestrating the starvation response [7,8,9]. NPF modulates feeding behavior through its receptor NPFR. Temperature preference shapes insect habitats and ranges: warm-climate *Drosophila melanogaster* avoids heat, whereas desert *Drosophila mojavensis* prefers high temperatures. [10]. Elevated temperatures accelerate insect metabolism, thereby influencing energy demand and feeding behavior [11]; for instance, in *Acyrthosiphon pisum*, protein consumption increased significantly under heat [12]. High-temperature stress markedly affects insect survival and reproduction, with heat shock proteins (HSPs) and related molecular chaperones occupying a central role in thermotolerance. By preserving protein stability and supporting immune defense, HSPs play an essential role in insect responses to both environmental and biotic stressors [13,14]. Insects adapt to low temperatures primarily through two strategies: freeze avoidance and freeze tolerance [15]. To mitigate cold-induced injury, they accumulate cryoprotectants such as glycerol and trehalose and deploy antifreeze proteins (AFPs) [16,17]. In addition, under cold conditions, insects adjust their metabolism to cope with cold stress [18]. Energy metabolism changes triggered by starvation or temperature stress often involve coordinated gene-level regulation, with adipokinetic hormone (*AKH*) and its receptor (*AKHR*) genes playing key roles.

AKH is widely distributed across diverse insect species [19], where it regulates lipid metabolism to supply energy for high-intensity activities. The AKH precursor comprises three functional domains: a signal peptide, a mature peptide, and an associated peptide [20,21]. AKHR is a typical G protein-coupled receptor (GPCR) characterized by high sequence similarity across insect species [22]. This structural conservation suggests the critical role of the AKH/AKHR signaling pathway in insect energy regulation. AKH is primarily secreted by the corpora cardiaca [23]. Upon release, AKH is transported via the hemolymph to the fat body, where it binds to AKHR, which is predominantly located on the surface of fat-body cell membranes [24,25]. AKH signaling is initiated by the specific interaction between the hormone and its receptor, AKHR [26]. In Coleopteran insects, AKH activates lipases, promoting the hydrolysis of triacylglycerols (TAG) into diacylglycerol (DAG) for energy supply [27]. In Dipteran species such as *Drosophila*, AKH primarily regulates the synthesis and release of trehalose [28].

The AKH/AKHR pathway has been extensively characterized as a core regulator of energy mobilization in diverse insect taxa, yet it remains unreported in the Chinese white pine beetle (*D. armandi*). *D. armandi* is a major pest of *Pinus armandii* in the Qinling–Bashan Mountains. It bores and feeds in the phloem, directly compromising host health, and carries symbiotic fungi that blue-stain the xylem, accelerating decline and often leading to tree death. Infestations can trigger cascading attacks by other pests, degrade forest ecosystems, and cause substantial economic losses [29]. Control in most affected areas still relies on traditional chemical measures, chiefly broad-spectrum insecticides, which carry risks of environmental contamination, resistance, and harm to non-target organisms. These limitations underscore an urgent need to elucidate the physiological and metabolic bases of adaptation and survival in *D. armandi* and to develop environmentally sustainable control strategies centered on RNA interference and key metabolic targets. To further explore this unaddressed question, our study elucidates the roles of *AKH* and *AKHR* in regulating energy metabolism in *D. armandi* under starvation, heat, and cold stress, providing mechanistic insight into its stress tolerance and a theoretical basis for more selective, ecologically compatible control.

## 2. Results

### 2.1. Identification and Characterization of DaAKH and DaAKHR Gene Sequences

The full-length sequences of *DaAKH* and *DaAKHR* from *D. armandi* were successfully cloned. The amino acid sequences of *DaAKH* and *DaAKHR* exhibited high similarity to the corresponding *DpAKH* and *DpAKHR* genes in *Dendroctonus ponderosae*, with identities of 93% and 97%, respectively (Table 1).

Full-length sequences of *DaAKH* and *DaAKHR* genes were obtained using RACE (rapid amplification of cDNA ends). The results showed that *DaAKH* encodes a 72-amino-acid protein, with a predicted molecular mass of 8.24 kDa and an isoelectric point (pI) of 5.22. *DaAKHR* encodes a 376-amino-acid protein, with a predicted molecular mass of 43.24 kDa and a pI of 9.33 (Table 2).

### 2.2. Structural and Functional Characteristics of DaAKH and DaAKHR

**Figure A1:**
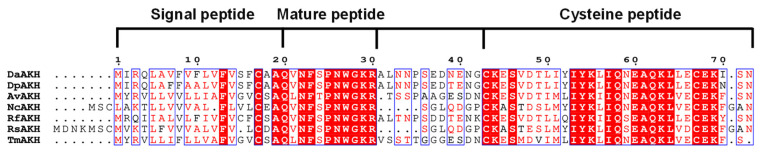
Multiple sequence alignment of *DaAKH* precursor amino acid sequence in *D. armandi. DpAKH* (Accession No.: ENN81145.1); *AvAKH* (Accession No.: RZB41086.1); *NcAKH* (Accession No.: AML80825.1); *RfAKH* (Accession No.: KAF7281926.1); *RsAKH* (Accession No.: AML80830.1); *TmAKH* (Accession No.: UXO98184.1). Sequence data were obtained from NCBI.

The amino acid sequence of the *DaAKH* precursor begins with a highly hydrophobic N-terminal segment, consistent with a classical signal peptide characteristic of secretory proteins. Immediately downstream of the signal peptide lies the putative mature AKH peptide. Its N-terminal residue is glutamine (Q), which serves as the precursor of pyroglutamate (pGlu), a modification frequently observed in active AKH peptides. The C-terminal end of the mature peptide contains tryptophan (W), a key residue implicated in receptor binding, followed by glycine (G) that acts as the amidation donor, and a pair of basic residues (KR) representing a typical proteolytic cleavage site. Following the mature peptide is a variable linker region that displays substantial sequence diversity among insect species and is presumed not to contribute directly to AKH bioactivity but may function in precursor folding and processing [21]. At the extreme C-terminus, a conserved cysteine-rich domain containing two cysteine residues is present; these cysteines are capable of forming an intramolecular disulfide bond, providing structural stability to the precursor. The AKH mature peptide and its cysteine-rich domain are highly conserved across insects, highlighting their evolutionary and functional significance. (Figure A1).

**Figure A2:**
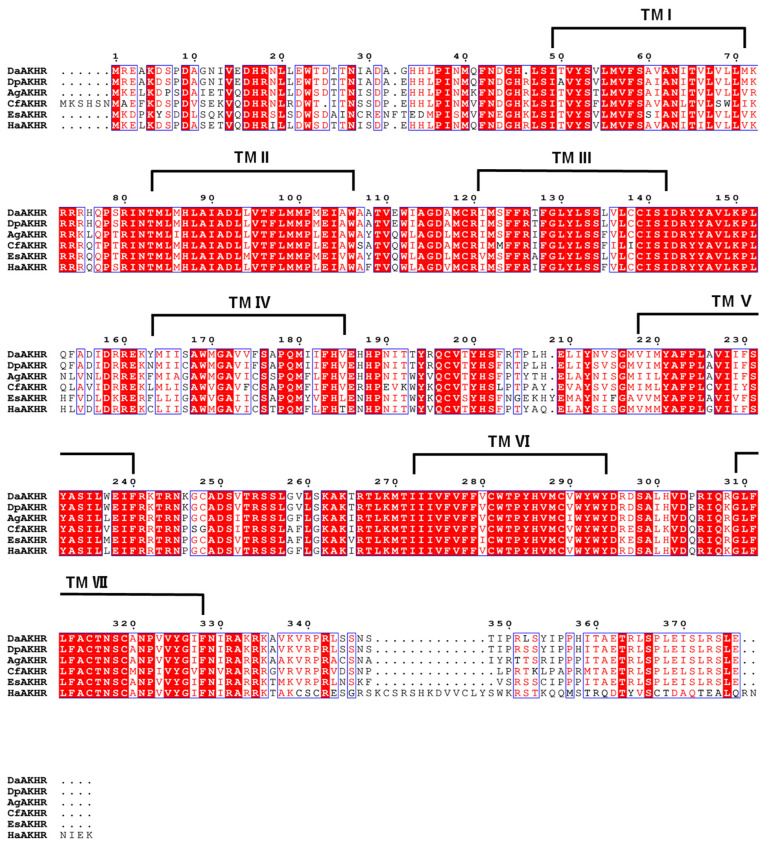
Multiple sequence alignment of *DaAKHR* amino acid sequence in *D. armandi. DpAKHR* (Accession No.: XP_048517977.1); *AgAKHR* (Accession No.: XP_050295995.1); *CfAKHR* (Accession No.: XP_060537041.1); *EsAKHR* (Accession No.: XP_066249755.1); *HaAKHR* (Accession No.: AVI00624.1). Sequence data were obtained from NCBI.

*DaAKHR* exhibits a canonical seven-transmembrane (TM I–VII) architecture with an extracellular N terminus, three intracellular loops, and an intracellular C-terminal tail, consistent with a class A G protein-coupled receptor (GPCR). The seven predominantly hydrophobic TM helices are embedded in the lipid bilayer and collectively form the ligand-binding cavity; ligand recognition is primarily mediated by the TM bundle together with the extracellular loops, with the N terminus likely contributing to auxiliary recognition and structural stabilization. Notably, a highly conserved DRY motif is located at the cytoplasmic end of TM III, serving as a hallmark “molecular switch” for receptor activation and G protein coupling in class A GPCRs. In addition, the intracellular C-terminal tail is enriched in serine/threonine residues, which likely serve as sites for GPCR kinase (GRK)-mediated phosphorylation. These topological and functional features are congruent with *DaAKH*-mediated signaling underlying lipid mobilization and provide a structural basis for mechanistic investigation (Figure A2).

### 2.3. Differential Expression of DaAKH and DaAKHR Across Developmental Stages and Tissues

**Figure 1:**
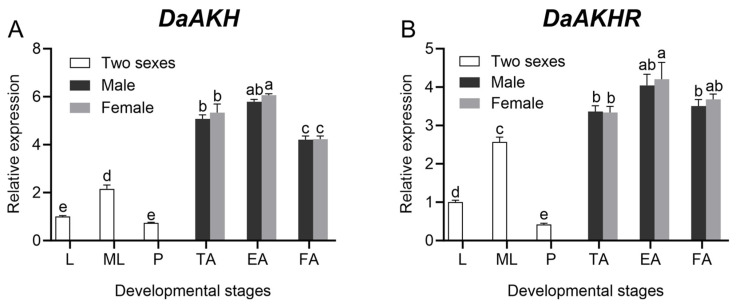
Expression levels of *DaAKH* and *DaAKHR* in *D. armandi* across distinct developmental stages. (**A**) Expression levels of *DaAKH* across developmental stages. (**B**) Expression levels of *DaAKHR* across developmental stages. Two sexes: sexually undifferentiated. L: larvae; ML: mature larvae; P: pupae; TA: teneral adult; EA: emerged adult; FA: feeding adult. Statistical differences in *DaAKH* and *DaAKHR* expression across developmental stages are denoted by superscript letters (*p* < 0.05, one-way ANOVA). All values are mean ± SE, *n* = 3.

*DaAKH* and *DaAKHR* were broadly expressed across all developmental stages of *D. armandi*. For *DaAKH*, among adults of *D. armandi*, expression was highest in emerged adults and lowest in feeding adults. No significant differences were observed between females and males within the same adult stage. Expression in larvae and pupae was significantly lower than in adults and mature larvae, with no difference between larvae and pupae (Figure 1A). For *DaAKHR*, within the same adult developmental stage of *D. armandi*, no significant sex-based differences were detected. However, emerged females exhibited significantly higher expression than feeding males and teneral adults of both sexes, while showing no significant difference relative to emerged males or feeding females. Expression in mature larvae was significantly higher than in larvae, and larvae exhibited significantly higher expression than pupae (Figure 1B).

**Figure 2:**
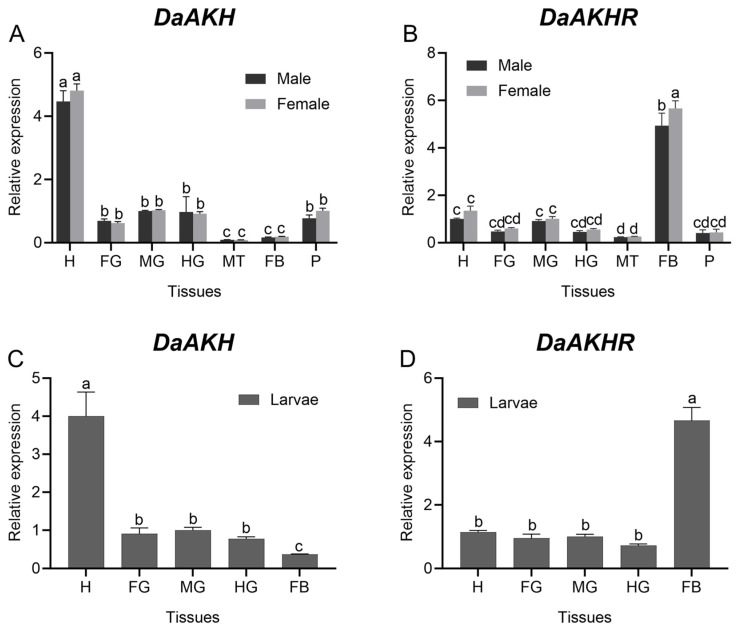
Expression levels of *DaAKH* and *DaAKHR* in different tissues of *D. armandi*. (**A**,**B**) Expression levels of *DaAKH* and *DaAKHR* across tissues in adult males and females. (**C**,**D**) Expression levels of *DaAKH* and *DaAKHR* across tissues in larvae. H: head; FG: foregut; MG: midgut; HG: hindgut; MT: malpighian tubule; FB: fat body; P: pheromone gland. Significant differences between *DaAKH* and *DaAKHR* among the development tissues are marked with letters (*p* < 0.05, one-way ANOVA). All values are mean ± SE, *n* = 3.

For *DaAKH* in adult *D. armandi*, expression in the head was highest in both females and males, followed by the foregut, midgut, hindgut, and pheromone gland, with no significant differences among gut regions and the pheromone gland. The lowest expression levels were detected in the malpighian tubule and fat body, with no significant difference between these two tissues. No sex-based differences in expression were observed in adults (Figure 2A). *DaAKH* was likewise predominantly expressed in the head of larvae, at significantly higher levels than in gut tissues. No significant differences in expression were detected among the foregut, midgut, and hindgut. Expression in the fat body was the lowest, significantly lower than in all other larval tissues (Figure 2C).

For *DaAKHR* in adult *D. armandi*, expression was highest in the fat body, with females exhibiting significantly higher levels than males. No significant differences were found among the head, foregut, midgut, hindgut, and pheromone gland, nor between sexes within these tissues. The malpighian tubule exhibited the lowest expression, significantly lower than in other adult tissues (Figure 2B). In larvae, *DaAKHR* expression was highest in the fat body, significantly exceeding that in all other larval tissues. No significant differences were detected among the head, foregut, midgut, and hindgut (Figure 2D).

### 2.4. Differential Expression of DaAKH and DaAKHR Under Starvation Stress

**Figure 3:**
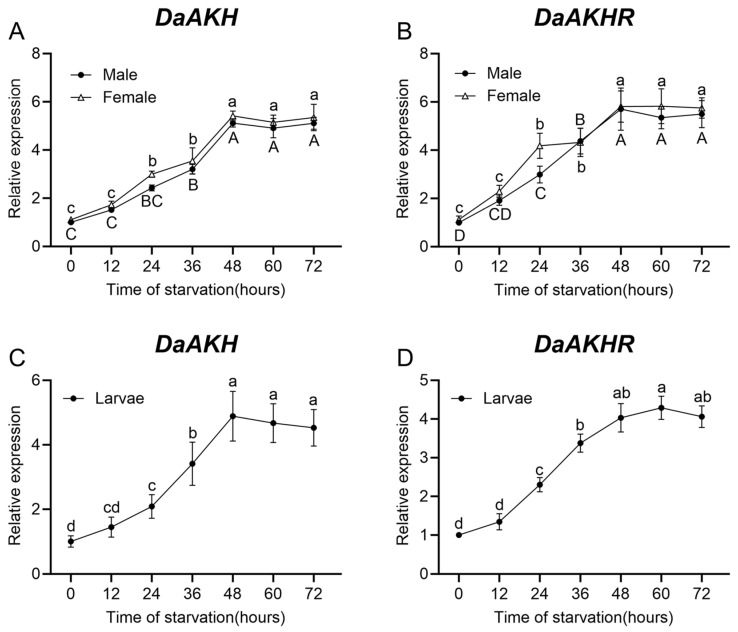
Relative expression levels of *DaAKH* and *DaAKHR* in *D. armandi* under starvation stress with varying durations. (**A**,**B**) Expression levels of *DaAKH* and *DaAKHR* across time points under starvation stress in adult males and females. (**C**,**D**) Expression levels of *DaAKH* and *DaAKHR* across time points under starvation stress in larvae. Significant differences between *DaAKH* and *DaAKHR* among the development tissues are marked with letters (*p* < 0.05, one-way ANOVA). Uppercase for male, lowercase for female adults and larvae. All values are mean ± SE, *n* = 3.

In larvae, female adults, and male adults of *D. armandi*, *DaAKH* expression increased significantly with prolonged starvation, reaching a sustained high level after 48 h. In larvae and female adults, expression at 24 h was significantly higher than at 0 h, whereas in male adults, a significant increase was observed at 36 h. No significant differences in expression were detected between 48 h and 72 h in any stage (Figure 3A,C).

*DaAKHR* exhibited a starvation-induced expression profile similar to that of *DaAKH*. In all stages (larvae, female adults and male adults), expression increased significantly at 24 h compared with 0 h. The highest expression levels occurred at 48 h in adult males and females, whereas larvae reached peak expression at 60 h. No significant differences in expression were observed between 48 h and 72 h across all stages (Figure 3B,D).

### 2.5. Heat Stress

#### 2.5.1. Determination of Heat Stress Temperature

**Figure 4:**
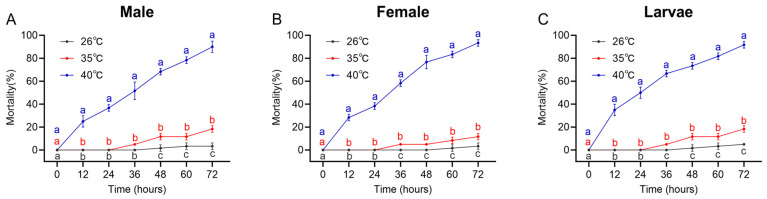
Mortality of *D. armandi* under different heat stress durations. (**A**–**C**) Mortality of males, females, and larvae under different heat stress durations. Significant differences in mortality among the three temperature treatments at each time point are marked with letters (*p* < 0.05, one-way ANOVA). Different colors represent different temperature treatments: gray for 26 °C, red for 35 °C, and blue for 40 °C. All values are mean ± SE, *n* = 3.

Heat-stress assays showed that at 40 °C, mortality in *D. armandi* larvae, female adults, and male adults exceeded 80%, which was significantly higher than at 35 °C and the 26 °C control. At 35 °C, mortality was higher than the control after 24 h and remained below 25% at 72 h. Accordingly, 35 °C was selected as the heat stress condition for subsequent experiments (Figure 4A–C).

#### 2.5.2. Differential Expression of *DaAKH* and *DaAKHR* Under Heat Stress

**Figure 5:**
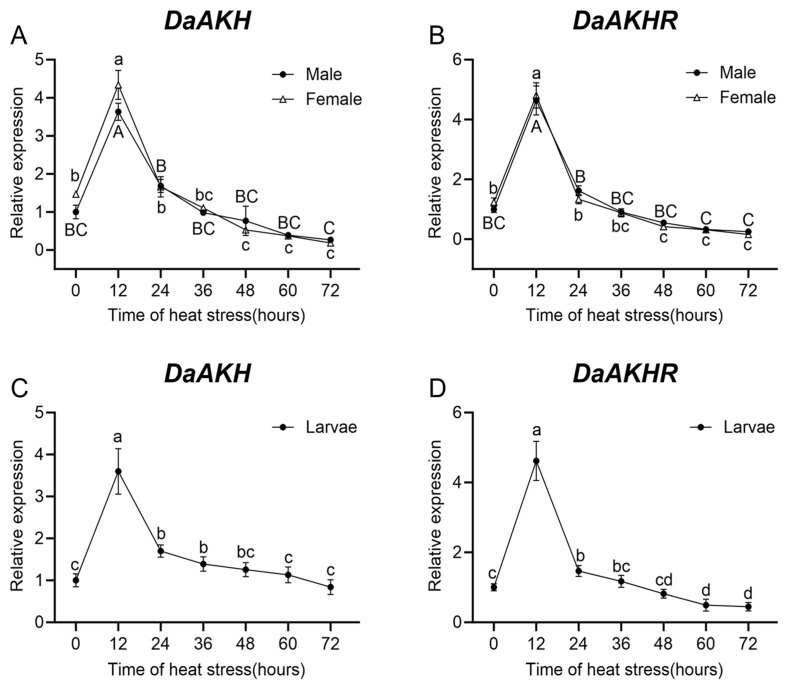
Relative expression levels of *DaAKH* and *DaAKHR* in *D. armandi* under heat stress with varying durations. (**A**,**B**) Expression levels of *DaAKH* and *DaAKHR* across time points under heat stress in adult males and females. (**C**,**D**) Expression levels of *DaAKH* and *DaAKHR* across time points under heat stress in larvae. Significant differences between *DaAKH* and *DaAKHR* among the development tissues are marked with letters (*p* < 0.05, one-way ANOVA). Uppercase for male, lowercase for female adults and larvae. All values are mean ± SE, *n* = 3.

Under heat stress, *DaAKH* expression in larvae, female adults, and male adults of *D. armandi* increased sharply within 12 h, reaching the highest level, which was significantly greater than at all other time points. Thereafter, expression declined to a relatively stable level. In male adults, no significant differences were detected between 24 h and 60 h, while female adults exhibited no significant variation between 36 h and 72 h. Larvae maintained stable expression between 24 h and 48 h and again between 48 h and 72 h (Figure 5A,C).

*DaAKHR* expression in larvae, female adults, and male adults was significantly elevated prior to 12 h of heat stress, followed by a sharp decline thereafter. In female adults, expression at 48 h was significantly lower than at 0 h, whereas male adults maintained levels that did not differ significantly from 0 h throughout the stress period. In larvae, expression at 60 h was significantly lower than at 0 h (Figure 5B,D).

### 2.6. Cold Stress

#### 2.6.1. Determination of Cold Stress Temperature

**Figure 6:**
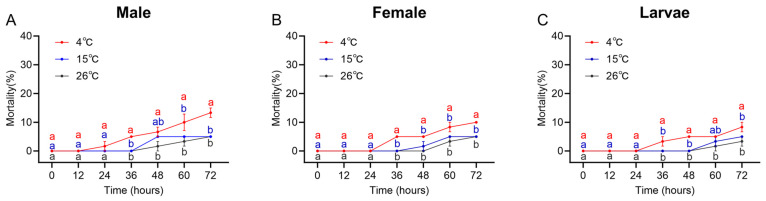
Mortality of *D. armandi* under different cold stress durations. (**A**–**C**) Mortality of males, females, and larvae under different cold stress durations. Significant differences in mortality among the three temperature treatments at each time point are marked with letters (*p* < 0.05, one-way ANOVA). Different colors represent different temperature treatments: red for 4 °C, blue for 15 °C, and gray for 26 °C. Comparative analysis of mortality rates at 26 °C under different exposure durations is presented in Table S1. All values are mean ± SE, *n* = 3.

Under cold stress, mortality in *D. armandi* larvae, female adults, and male adults at 15 °C did not differ significantly from that at the 26 °C control. At 4 °C, male adults exhibited increased mortality at 12 h, while larvae and female adults showed elevated mortality at 24 h. At 72 h, mortality across all three developmental stages remained below 15%, meeting the requirements for subsequent experiments. Accordingly, 4 °C was selected as the cold stress condition for this study (Figure 6A–C).

#### 2.6.2. Differential Expression of *DaAKH* and *DaAKHR* Under Cold Stress

**Figure 7:**
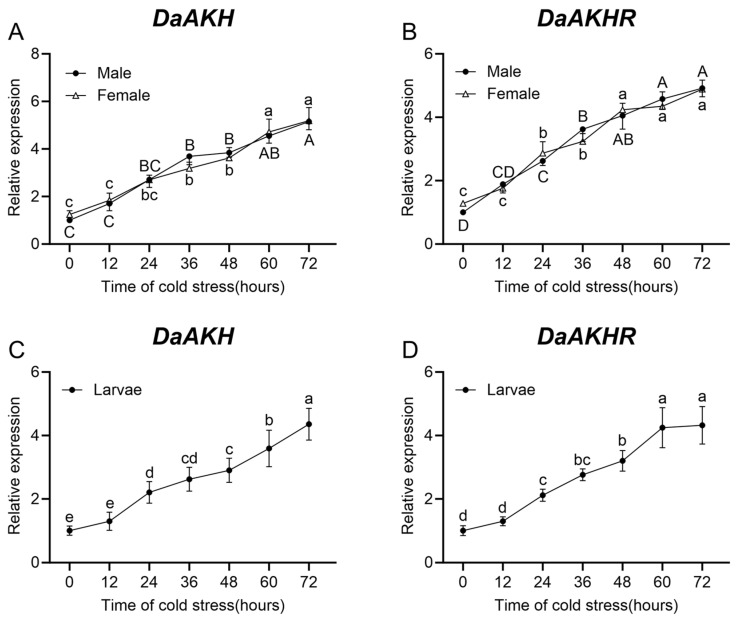
Relative expression levels of *DaAKH* and *DaAKHR* in *D. armandi* under cold stress with varying durations. (**A**,**B**) Expression levels of *DaAKH* and *DaAKHR* across time points under cold stress in adult males and females. (**C**,**D**) Expression levels of *DaAKH* and *DaAKHR* across time points under cold stress in larvae. Significant differences between *DaAKH* and *DaAKHR* among the development tissues are marked with letters (*p* < 0.05, one-way ANOVA). Uppercase for male, lowercase for female adults and larvae. All values are mean ± SE, *n* = 3.

Under cold stress, *DaAKH* expression in larvae, female adults, and male adults of *D. armandi* increased progressively over time. In male adults, no significant differences were detected between 24 h and 60 h; in female adults, no significant variation was observed between 24 h and 48 h; and in larvae, expression did not change significantly between 24–36 h and 36–48 h (Figure 7A,C).

*DaAKHR* expression likewise increased with the duration of cold stress in larvae, female adults, and male adults. In both female and male adults, expression showed no significant change after 48 h, whereas in larvae, *DaAKHR* levels remained stable after 60 h (Figure 7B,D).

### 2.7. RNAi-Based Analysis of DaAKH and DaAKHR Functions in D. armandi

#### 2.7.1. RNAi Efficiency for *DaAKH* and *DaAKHR*

**Figure 8:**
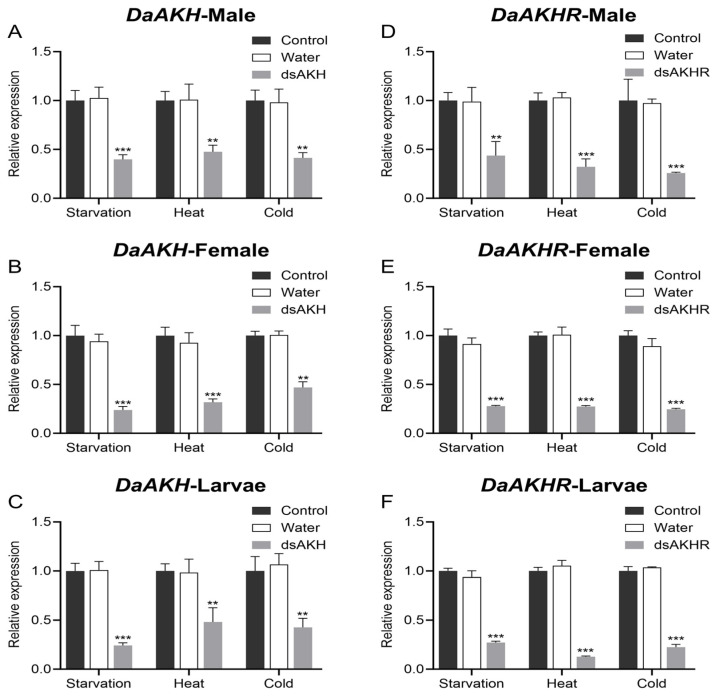
Relative expression levels of *DaAKH* and *DaAKHR* at 72 h post-RNAi. (**A**–**C**) Relative expression levels of *DaAKH* at 72 h post-RNAi treatment in adult males, adult females, and larvae. (**D**–**F**) Relative expression levels of *DaAKHR* at 72 h post-RNAi treatment in adult males, adult females, and larvae. Control: untreated group; Water: DEPC-treated water. The asterisk indicates a significant difference between dsRNA treatment and control groups. (** *p* ≤ 0.01, *** *p* ≤ 0.001, one-way ANOVA). All values are mean ± SE, *n* = 3.

Under starvation, heat, and cold stress, larvae, female adults, and male adults of *D. armandi* were injected with dsRNA, and *DaAKH* and *DaAKHR* transcript levels were assessed after 72 h. Injection of dsAKH significantly reduced *DaAKH* expression in all developmental stages under all three stress conditions (Figure 8A–C). Similarly, dsAKHR administration markedly decreased *DaAKHR* transcript abundance under starvation, heat, and cold stress (Figure 8D–F). These findings confirm that RNAi effectively suppresses *DaAKH* and *DaAKHR* expression across diverse stress contexts.

#### 2.7.2. Mortality of *D. armandi* Under Different Stresses Following RNAi

**Figure 9:**
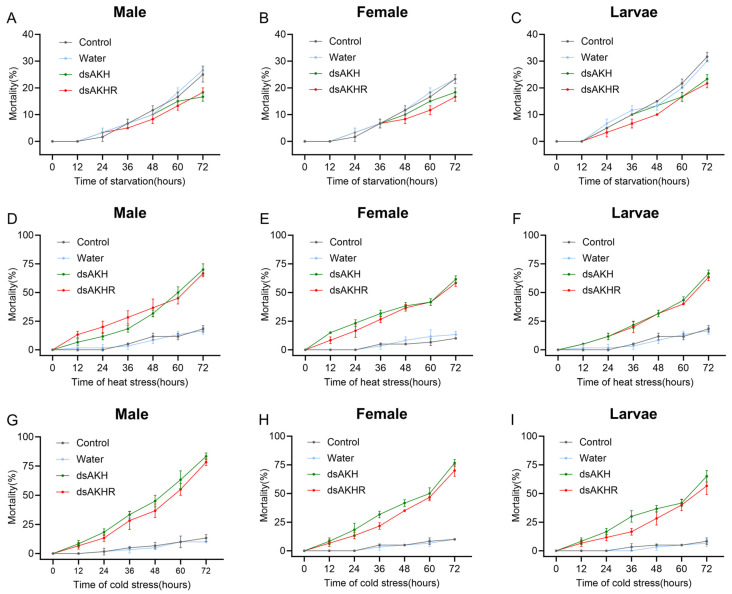
Mortality of *D. armandi* under different stresses following RNAi of *DaAKH* and *DaAKHR* at varying durations. (**A**–**C**) Mortality in adult males, adult females, and larvae under starvation stress following RNAi of varying durations. (**D**–**F**) Mortality in adult males, adult females, and larvae under heat stress following RNAi of varying durations. (**G**–**I**) Mortality in adult males, adult females, and larvae under cold stress following RNAi of varying durations. The RNA interference treatment was administered at different time points (0 h, 12 h, 24 h, 36 h, 48 h, 60 h and 72 h). Differences between the treatment and control groups at each exposure time point are detailed in Supplementary Table S2 and are indicated by letters (*p* < 0.05, one-way ANOVA) in Table S2. All values are presented as mean ± SE, *n* = 3.

Under starvation stress for 72 h, larvae, female adults, and male adults injected with dsAKH or dsAKHR exhibited lower mortality than both untreated and DEPC-treated controls. Notably, mortality in dsAKHR-injected females and dsAKH-injected males was only 17%. Among the treated groups, larvae consistently showed higher mortality than adults, with mortality rates of 23% and 22% following dsAKH and dsAKHR injection, respectively (Figure 9A–C).

Under heat stress, mortality in all dsAKH- or dsAKHR-injected larvae and adults was significantly elevated. The highest mortality (70%) was recorded in dsAKH-treated males after 72 h, whereas dsAKHR-treated females exhibited comparatively lower mortality (58%) under the same conditions (Figure 9D–F).

Under cold stress, RNAi markedly increased mortality in larvae, female adults, and male adults. Larvae exhibited lower mortality than females, whereas dsRNA treatment in males resulted in the highest mortality (80%) after 72 h exposure. Across all stages, mortality following dsAKH injection exceeded that observed with dsAKHR under cold stress (Figure 9G–I).

#### 2.7.3. Effects of RNAi on Energy Metabolism in *D. armandi* Under Starvation Stress

**Figure 10:**
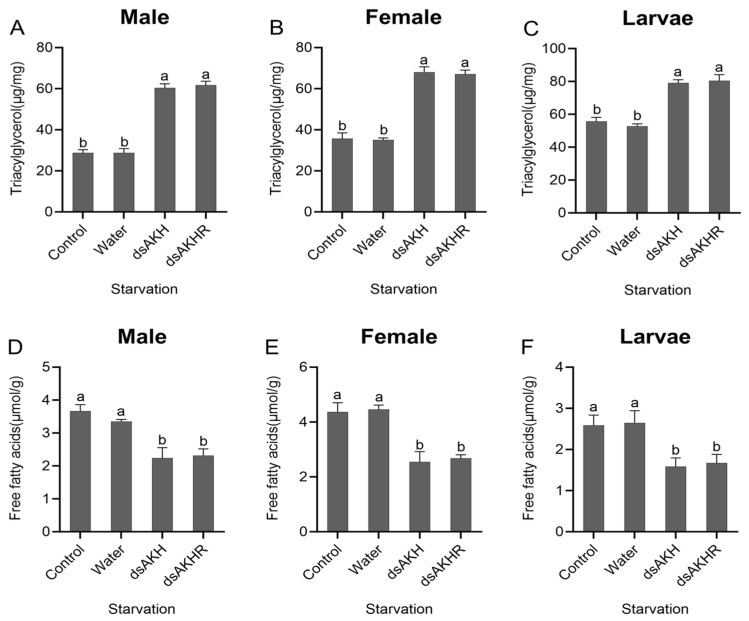
Changes in lipid metabolism under starvation stress at 72 h post-RNAi. (**A**–**C**) Triacylglycerol levels in adult males, adult females, and larvae under starvation stress. (**D**–**F**) Free fatty acid levels in adult males, adult females, and larvae under starvation stress. Statistical differences among treatments are indicated by different superscript letters (*p* < 0.05, one-way ANOVA). All values are expressed as mean ± SE, *n* = 3.

Larvae, female adults, and male adults of *D. armandi* injected with dsAKH or dsAKHR under starvation stress showed a significant increase in TAG content compared with controls. In both RNAi treatments, TAG levels in males increased by approximately 50% (Figure 10A–C). Conversely, free fatty acid (FFA) content was markedly reduced, with more than a 50% decline detected in larvae, females, and males across both treatments (Figure 10D–F). Within the treated groups, no significant differences in TAG or FFA content were observed between dsAKH and dsAKHR injections for any developmental stage. Interference with *DaAKH* and *DaAKHR* under starvation stress reduced lipid metabolism, leading to lipid accumulation. The reduced mortality following interference under starvation suggests that dsAKH or dsAKHR injection diminished lipid metabolism and promoted lipid storage, thereby slowing energy depletion and alleviating starvation-induced mortality.

**Figure 11:**
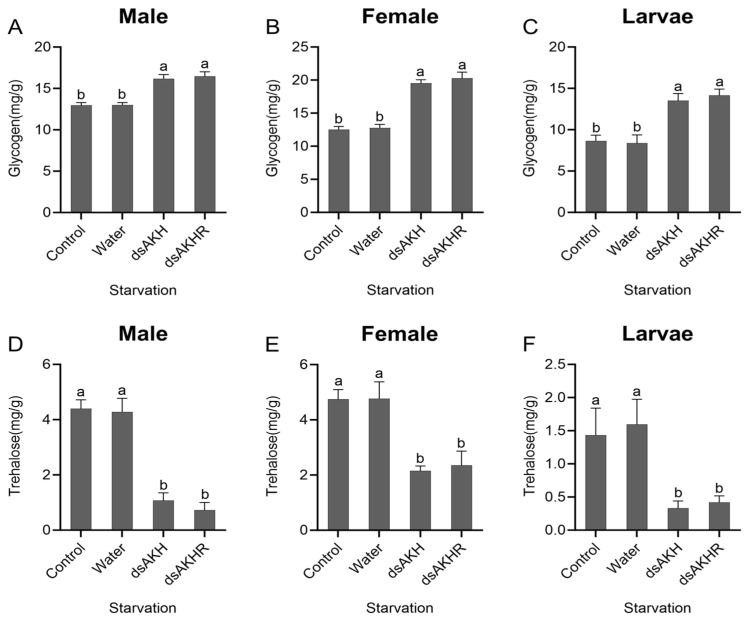
Changes in carbohydrate metabolism of *D. armandi* under starvation stress at 72 h post-RNAi. (**A**–**C**) Glycogen levels in adult males, adult females, and larvae under starvation stress. (**D**–**F**) Trehalose levels in adult males, adult females, and larvae under starvation stress. Statistical differences among treatments are indicated by different superscript letters (*p* < 0.05, one-way ANOVA). All values are expressed as mean ± SE, *n* = 3.

Injection of dsAKH or dsAKHR into larvae, female adults, and male adults led to a significant increase in glycogen content, with larvae exhibiting the largest rise (87%) under both RNAi treatments (Figure 11A–C). Trehalose content was significantly reduced; declines in larvae and females exceeded those in males, and larval trehalose decreased by 80% in both treatments (Figure 11D–F). No significant differences were detected between dsAKH and dsAKHR for either glycogen or trehalose. Under starvation stress, interference with *DaAKH* and *DaAKHR* impaired glycogen breakdown, resulting in glycogen accumulation. The decrease in trehalose content suggests that, when lipid metabolism is reduced and glycogen breakdown is inhibited, trehalose catabolism provides an alternative energy source to cope with starvation stress.

#### 2.7.4. Effects of RNAi on Energy Metabolism in *D. armandi* Under Heat Stress

**Figure 12:**
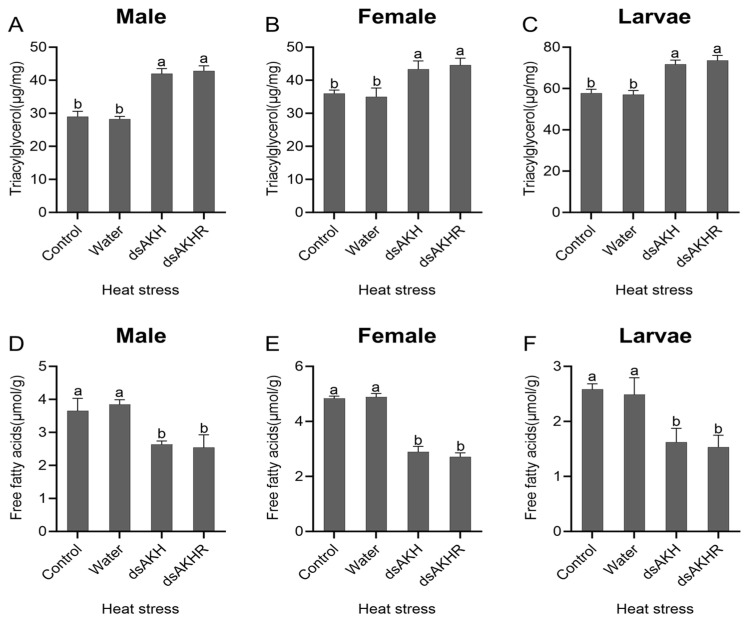
Changes in lipid metabolism of *D. armandi* under heat stress at 72 h post-RNAi. (**A**–**C**) Triacylglycerol levels in adult males, adult females, and larvae under heat stress. (**D**–**F**) Free fatty acid levels in adult males, adult females, and larvae under heat stress. Statistical differences among treatments are indicated by different superscript letters (*p* < 0.05, one-way ANOVA). All values are expressed as mean ± SE, *n* = 3.

Under heat stress, larvae, female adults, and male adults of *D. armandi* injected with dsAKH or dsAKHR exhibited a significant increase in TAG content; males showed the greatest rise, with TAG increasing by 40% in both RNAi groups (Figure 12A–C). FFA levels were significantly reduced (Figure 12D–F). No significant differences were observed between dsAKH and dsAKHR. Under heat stress, interference for 72 h suppressed lipid metabolism, manifested by lipid accumulation and decreased levels of lipid-derived metabolites.

**Figure 13:**
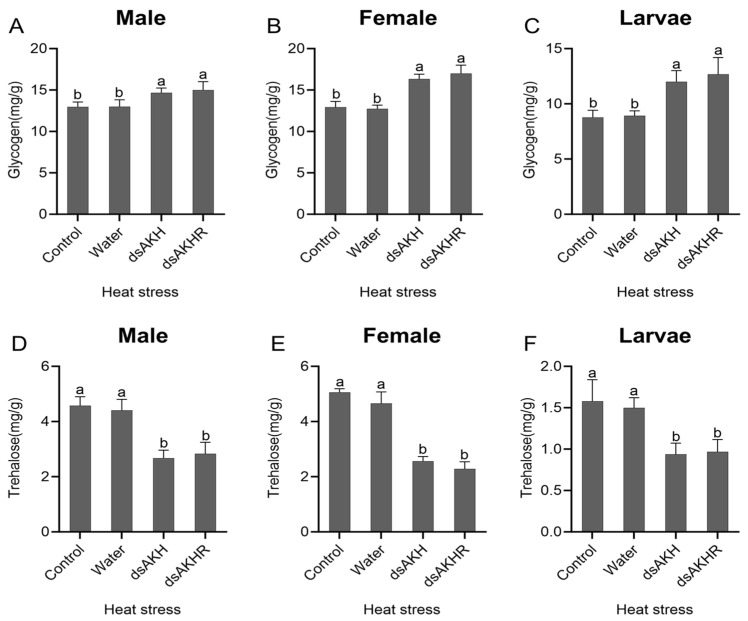
Changes in carbohydrate metabolism of *D. armandi* under heat stress at 72 h post-RNAi. (**A**–**C**) Glycogen levels in adult males, adult females, and larvae under heat stress. (**D**–**F**) Trehalose levels in adult males, adult females, and larvae under heat stress. Statistical differences among treatments are indicated by different superscript letters (*p* < 0.05, one-way ANOVA). All values are expressed as mean ± SE, *n* = 3.

Glycogen measurements under heat stress following RNAi revealed significant increases across larvae, females, and males, with larvae injected with dsAKHR showing the largest elevation (Figure 13A–C). Trehalose content decreased significantly after RNAi, with reductions of 40–50% in larvae, females, and males in both dsAKH and dsAKHR groups (Figure 13D–F). No significant differences were detected between dsAKH and dsAKHR. Under heat stress, interference suppressed glycogen breakdown, leading to glycogen accumulation. The post-interference decline in trehalose likely reflects reduced biosynthesis, potentially compromising heat-stress tolerance.

#### 2.7.5. Effects of RNAi on Energy Metabolism in *D. armandi* Under Cold Stress

**Figure 14:**
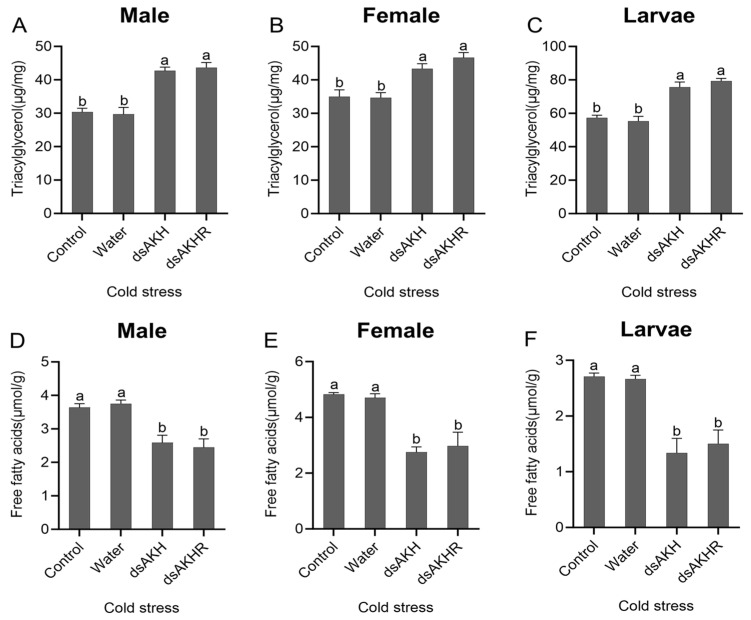
Changes in lipid metabolism of *D. armandi* under cold stress at 72 h post-RNAi. (**A**–**C**) Triacylglycerol levels in adult males, adult females, and larvae under cold stress. (**D**–**F**) Free fatty acid levels in adult males, adult females, and larvae under cold stress. Statistical differences among treatments are indicated by different superscript letters (*p* < 0.05, one-way ANOVA). All values are expressed as mean ± SE, *n* = 3.

Under cold stress, lipid metabolic changes in *D. armandi* following dsAKH or dsAKHR injection closely resembled those observed under heat stress. TAG content increased significantly, indicating lipid accumulation, with males showing the largest elevation—approximately 40% in both RNAi treatments (Figure 14A–C). FFA levels were markedly reduced, with larvae exhibiting the greatest decline—about 50% in both dsAKH and dsAKHR groups (Figure 14D–F). No significant differences were observed between dsAKH and dsAKHR. Under cold stress, RNA interference suppressed lipid metabolism in *D. armandi*, thereby diminishing cold-stress tolerance.

**Figure 15:**
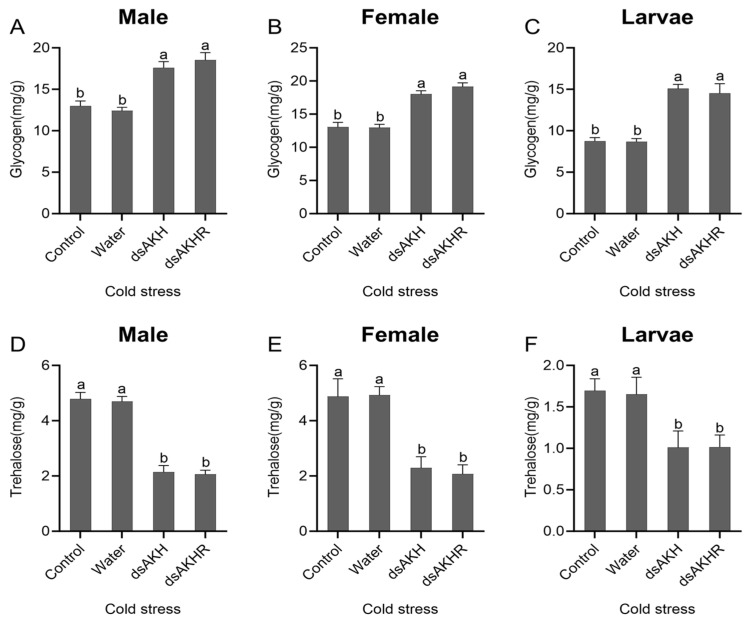
Changes in carbohydrate metabolism of *D. armandi* under cold stress at 72 h post-RNAi. (**A**–**C**) Glycogen levels in adult males, adult females, and larvae under cold stress. (**D**–**F**) Trehalose levels in adult males, adult females, and larvae under cold stress. Statistical differences among treatments are indicated by different superscript letters (*p* < 0.05, one-way ANOVA). All values are expressed as mean ± SE, *n* = 3.

Glycogen levels rose significantly after RNAi, with larvae showing the highest increase, reaching 70% in both treatments (Figure 15A–C). Trehalose measurements revealed a substantial decrease in both female and male adults after RNAi for both dsAKH and dsAKHR (Figure 15D–F). No significant differences were detected between the two RNAi groups. We observed glycogen accumulation under cold stress following RNA interference, consistent with inhibited glycogenolysis. Because trehalose functions as a major cryoprotectant in insects, its reduction under cold stress after RNAi likely reflects weakened cold tolerance.

## 3. Discussion

We cloned and characterized the full-length sequences of the *AKH* gene (*DaAKH*) and its receptor gene (*DaAKHR*) from *D. armandi*. Both sequences display the canonical features of AKH and AKHR and are highly similar to the corresponding genes (*DpAKH* and *DpAKHR*) in *D. ponderosae*. AKH peptides are highly conserved [30]. For example, *Manduca sexta* and *B*. *mori* share identical segments within the mature AKH peptide [31,32]. The *DaAKH* mature peptide contains an N-terminal Q (pGlu), a C-terminal W followed by G for amidation and KR for proteolytic cleavage, and a cysteine-rich C-terminal domain forming an intramolecular disulfide. *DaAKHR* is a canonical class A GPCR with seven transmembranes, an intact DRY motif, and a Ser/Thr-rich C-terminal tail. It is noteworthy that most coleopteran pest species do not carry the prototypical coleopteran AKH isoform [33]. This divergence may reflect a coevolutionary response of AKH to host-tree secondary metabolites, suggesting a dual role in energy metabolism and detoxification pathways, with important implications for sustainable pest management.

We found that *DaAKH* is highly expressed in the head, whereas *DaAKHR* shows the highest expression in the fat body. This distribution pattern is consistent with *Coccinella septempunctata* [34], underscoring the generality of AKH-mediated regulation across insects. As a regulator of lipid metabolism in the migratory locust, *Locusta migratoria*, the AKH-related peptide (ACP) maintains TAG homeostasis by modulating lipid metabolic genes [35]. Likewise, in the fat body of *L. migratoria*, TAG lipase is under AKH control to support flight metabolism [36]. In *Leptinotarsa decemlineata*, an endogenous AKH-family octapeptide (Melme-CC) stimulates lipase activation [37]. Furthermore, in *B. mori*, three AKH-related peptides (AKH1, AKH2, ACP) and three receptors (AKHR1, AKHR2a, AKHR2b) are present, with AKH1 and AKH2 regulating energy homeostasis via the AKHR1 receptor [38].

Lipid reserves are preferentially mobilized during starvation [4]. Consistent with this physiological role, AKH/AKHR constitute a bioactive signaling axis embedded within the neuroendocrine network [39]. Under starvation, AKH signaling mobilizes lipids via AKHR and modulates starvation-induced locomotor activity [40]. In *Drosophila*, ILPs and AKH are key hormonal regulators of metabolic homeostasis [41,42]. Under starvation, *DaAKH* and *DaAKHR* transcript levels surged to a peak within 48 h and then stabilized, suggesting that they mobilize stored energy to sustain survival in early starvation, whereas energy conservation during prolonged starvation may enhance survival. In line with this, *AKH* and *AKHR* expression are likewise upregulated after 48 h of starvation in *Nilaparvata lugens* [43]. Additional lines of evidence converge on this mechanism: in *Drosophila*, *AKH* mutants exhibit markedly reduced trehalose and a decreased metabolic rate [44,45]; in *Gryllus bimaculatus*, RNAi against *AKHR* lowers trehalose while significantly elevating TAG [46]; in *C. septempunctata*, deletion of *AKH* or *AKHR* increases TAG [34]; in *Bactrocera dorsalis*, *AKHR* knockdown raises TAG [47]; and in *Panonychus citri*, *AKH* silencing disrupts lipid metabolism and carbohydrate homeostasis [48]. In *D. melanogaster*, *AKH* deficiency confers starvation resistance owing to lipid accumulation [49]. Similarly, *AKHR* loss-of-function mutants of *Drosophila* display excessive lipid accumulation, defects in glycogen metabolism, and prolonged survival under starvation [50]. Consistent with these patterns, in *D. armandi*, we observed that dsRNA targeting *DaAKH* or *DaAKHR* reduces mortality under starvation. After RNAi, starving *D. armandi* accumulated lipids, with TAG markedly increased and FFA significantly decreased. Taken together, our findings demonstrate that *DaAKH* and *DaAKHR* participate in the regulation of lipid metabolism under starvation, facilitating rapid adaptation to resource scarcity. When their signaling is suppressed by dsRNA, systemic metabolism is dampened, lipids and glycogen accumulate, and mortality during starvation is paradoxically reduced. At the neuropeptide level, NPF/sNPF in *D. armandi* has been shown to directly influence feeding and body weight [8,9]. In light of the role of NPF/NPFR in driving feeding behavior [7], we hypothesize synergistic or antagonistic cross-pathway regulation between the AKH/AKHR and the NPF/sNPF network. Under starvation, AKH/AKHR signaling is thought to be upregulated to mobilize energy reserves, while NPF/sNPF likely adjusts feeding behavior to match metabolic needs.

Heat stress reshapes lipid metabolism in insects; for example, lipid content declines significantly at elevated temperatures in *Tenebrio molitor* [51]. Heat stress in this study triggered a rapid upregulation of *DaAKH* and *DaAKHR* expression within 12 h. Expression then rapidly decreased, consistent with cumulative heat injury under prolonged exposure and a consequent depression of metabolism. Under heat stress, dsRNA targeting *DaAKH* and *DaAKHR* significantly increased mortality, and TAG and glycogen levels were elevated, suggesting reduced energy mobilization and increased storage accumulation following interference. This suppression, together with the physiological impact of heat stress, may have contributed to the observed rise in mortality. HSP induction represents a fundamental molecular mechanism underlying thermotolerance [14]. Because HSP chaperone activity requires a stable energy supply, we speculate that AKH/AKHR contributes to heat stress adaptation by maintaining metabolic homeostasis, thereby creating favorable conditions for effective protein folding and proteostasis. The changes in *DaAKH* and *DaAKHR* expression under heat stress observed in our study mirror this proposed mechanism, supporting their functional role as a rapid metabolic “switch” that facilitates coping with thermal challenges.

Under cold stress, insects enhance survival by lowering metabolic rate and accumulating cryoprotectants such as trehalose and glycerol [52]. In *T. molitor*, larval cold storage leads to total lipid increases [53]. Larvae of *D. armandi* reduce respiration during overwintering [54]. In larvae of *D. ponderosae*, glycerol and proline rise markedly in autumn as temperatures decline [55]. In *Ips typographus*, hemolymph trehalose increases in autumn [56]. Cold stress in insects entails differential regulation of numerous genes at the transcriptional, translational, and post-translational levels [52]. The protective role of AFPs under cold stress has been demonstrated. In overwintering larvae of *D. armandi*, RNA interference targeting *AFP* led to a marked increase in low-temperature mortality [17]. Under cold stress, *DaAKH* and *DaAKHR* transcript levels in adult males and females increased steadily, whereas *DaAKHR* of larvae reached a plateau after 60 h. This divergence likely reflects stage-specific cold-adaptation strategies: adults sustain energy mobilization to support thermoregulation, while larvae achieve long-term cold tolerance through metabolic homeostasis. Trehalose, the principal circulating sugar in insect hemolymph, plays dual roles under thermal stress, serving both as an energy reserve and a protein stabilizer [57,58]. Consistent with these dynamics, RNAi-treated *D. armandi* exhibited higher mortality than controls and significantly lower trehalose under cold stress, demonstrating that interference with *DaAKH* and *DaAKHR* compromises energy metabolism and cold acclimation. AKH mobilizes lipids and glycogen and maintains hemolymph carbohydrate balance, thereby supporting basal energy supply and enhancing cold tolerance [59]. In *Apis mellifera*, cold stress elevates *AKHR* transcript levels in larvae [60]. Latest research indicates that AKHR-mediated signaling acts as an endocrine sentinel to promote survival under cold stress via metabolic modulation [61]. Our results show that *D. armandi* upregulates *DaAKHR* expression during cold exposure, supporting a conserved role of AKHR in maintaining energy homeostasis and enhancing cold tolerance across insect taxa.

AKH also modulates energy metabolism via nutrient sensing. Existing studies have shown that, in *D. melanogaster*, AKH secretion under low-energy states involves K^+^ and Ca^2+^ channels and is triggered by AMPK signaling [39]. In *Drosophila*, AKH-producing neuroendocrine cells (APCs) directly sense intracellular ATP and changes in sugar concentration and dynamically modulate hormone release [62]. Under energy stress, APCs release AKH to mobilize stored glycogen and lipids, converting them into usable energy to maintain metabolic homeostasis [63]. Specific amino acids activate APCs, eliciting Ca^2+^-dependent AKH release to promote lipolysis; AKH also regulates calcium wave dynamics in the fat body through developmental stage-specific mechanisms, shaping lipolysis and adaptation to starvation [64].

**Figure 16:**
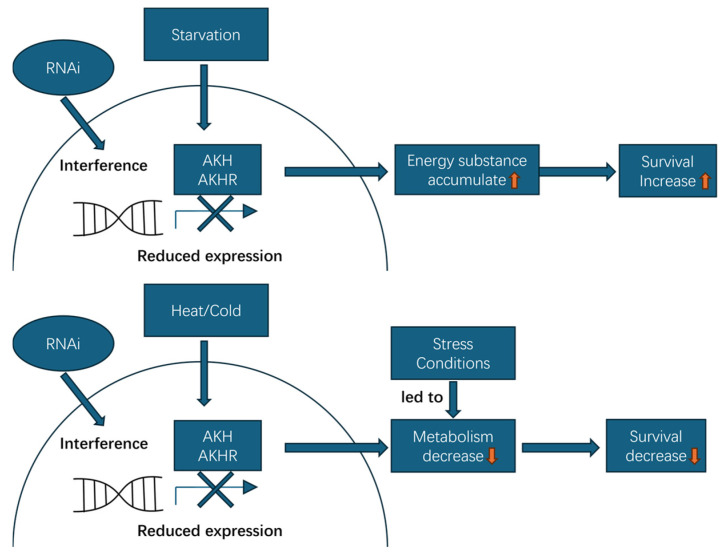
Schematic model of the effects of AKH/AKHR RNA interference on stress responses in *D. armandi*. Under starvation, silencing AKH or AKHR attenuates metabolic activity and promotes accumulation of energy stores, thereby conserving energy and reducing starvation-induced mortality. In contrast, under heat or cold stress, AKH/AKHR knockdown suppresses metabolic mobilization, compromises stress tolerance, and increases mortality.

To ensure precise and reproducible dsRNA delivery, we used microinjection. This method enables strict control of dosage, avoids dsRNA degradation in the digestive tract and feeding-related variability, and is a widely validated approach for RNAi in coleopteran insects. No significant differences were detected between the DEPC-treated water-injected group and the sham control in mortality, baseline metabolic profiles, or *DaAKH*/*DaAKHR* expression levels. These results confirm that mechanical injury did not act as a confounding variable and that the stress-dependent bidirectional survival phenotypes aligned with gene knockdown efficiency. Our study demonstrates that *DaAKH* and *DaAKHR* in *D. armandi* participate in the regulation of energy metabolism under different stress conditions (Figure 16). Future work should further delineate the molecular regulatory network of the AKH/AKHR signaling pathway in *D. armandi* and elucidate the specific mechanisms underlying environmental adaptation. Particular attention should be paid to how AKH/AKHR signaling modulates glycogen and lipid metabolism through downstream transcription factors (e.g., CREB) in a tissue-specific manner, such as in the fat body and neurons [65]. Future studies should also investigate how upstream nutrient-sensing neurons (e.g., capaergic neurons) integrate fat body activities by inhibiting AKH release to maintain systemic metabolic homeostasis under environmental stress [66]. RNAi experiments indicate that *AKH* and *AKHR* serve as pivotal regulators in responses to environmental stress. Building on this, integrating nanocarrier-mediated delivery of dsRNA [67] with temperature- or starvation-induced gene expression patterns may enable the development of environmentally responsive, highly targeted biopesticides [68]. Under defined ecological conditions, this strategy could precisely disrupt pest metabolic pathways, induce metabolic dysregulation, and thereby achieve the dual goals of green control and ecological safety.

## 4. Materials and Methods

### 4.1. Experimental Insects

Specimens of *D. armandi* were collected in the Huoditang Teaching and Experimental Forest, Ningshan County, Shaanxi Province, in the central Qinling Mountains (33°18′–33°28′ N, 108°21′–108°39′ E). Larvae and pupae were obtained by sequential bark removal: the phloem–xylem boundary was incised to expose galleries, and individuals at different developmental stages were collected.

### 4.2. Gene Cloning and Sequence Analysis

Total RNA was extracted from *D. armandi* using a UNIQ-10 column-based Trizol Total RNA Extraction Kit (Sangon Biotech, Shanghai, China). First-strand cDNA was synthesized with the FastKing First-Strand cDNA Synthesis Kit (Tiangen, Beijing, China). Based on conserved *AKH* and *AKHR* gene sequences from Coleopteran insects retrieved from the NCBI database, primers targeting *DaAKH* and its receptor *DaAKHR* were designed using Primer Premier 5.0 (Table S3). Specific 3′ and 5′ RACE primers were designed from the obtained sequences (Table S4), and full-length sequences were amplified using the SMARTer™ RACE cDNA Amplification Kit (Clontech Laboratories Inc., Mountain, CA, USA). All fragments were assembled with DNAMAN 6.0 software, and full-length primers were designed for sequence verification (Table S5). The complete sequences were analyzed by BLASTP against entries in GenBank for comparative purposes.

The molecular weight and isoelectric point of the proteins encoded by *DaAKH* and *DaAKHR* were predicted using ProtParam tool (http://web.expasy.org/protparam/, accessed on 5 March 2024). N-terminal signal peptides were predicted using SignalP 4.1 (SignalP 4.1—DTU Health Tech—Bioinformatic Services). The presence and topology of transmembrane domains were predicted using the TMHMM Server v.2.0 (https://services.healthtech.dtu.dk/services/TMHMM-2.0/, accessed on 10 March 2024).

### 4.3. Treatment of Insects

#### 4.3.1. Insect Sampling at Different Developmental Stages and Tissues

*D. armandi* were categorized into larvae, mature larvae, pupae, teneral adult, emerged adult, and feeding adult. Each developmental stage was treated as one experimental group, with three biological replicates per group; each replicate comprised three individuals. Total RNA extraction from whole bodies and cDNA synthesis across developmental stages were performed as described above. Adults and larvae were dissected under an SZX16 stereomicroscope (Olympus, Tokyo, Japan). In adults, the head, foregut, midgut, hindgut, malpighian tubules, and fat body were collected; in larvae, the head, foregut, midgut, hindgut, and fat body were collected. Dissected tissues were snap-frozen in liquid nitrogen and stored at −80 °C for long-term preservation. Three biological replicates were prepared for each experimental group. Total RNA extraction and cDNA synthesis from each tissue followed the procedures described above.

#### 4.3.2. Life-Stage Selection for Stress Assays

Pupae were excluded from stress assays because they are non-feeding, immobile, and depend primarily on larval energy reserves, making AKH/AKHR-mediated short-term metabolic adjustments unlikely. Moreover, their delicate integument and sensitivity to temperature shifts reduce survival and reproducibility. We therefore focused on larvae and adults, which show active metabolism and greater responsiveness to environmental stress.

#### 4.3.3. Setting of Stresses

Stress treatments were conducted under both heat and cold conditions. For heat stress, 26 °C was used as the control. Based on the finding that adult *Araecerus fasciculatus* exhibit 100% mortality after 12 h of exposure to 45 °C [69] and considering high-temperature gradient stress experiments in aphids [70], two heat treatments were established at 35 °C and 40 °C. Male and female emerged adults, as well as larvae, were divided into three groups and placed into glass Petri dishes containing fresh *P. armandii* phloem as food. Individuals were exposed to the temperature treatments for 12, 24, 36, 48, 60, and 72 h. Mortality rates at each time point were recorded for the control (26 °C) and both heat treatment groups using freshly collected *D. armandi* as baseline; the optimal temperature for heat stress assays was subsequently determined. For cold stress, a similar gradient was applied, with 26 °C as control. Based on the gradient of cold stress for *D. armandi* larvae, two temperature conditions (15 °C and 4 °C) were established [17,54,71]. Exposure durations and procedures were identical to those in the heat stress experiment, and mortality rates were assessed to determine the optimal temperature for cold stress assays.

Male and female dispersing adults and larvae were divided into three groups and placed in glass Petri dishes. Starvation assays were performed for 12, 24, 36, 48, 60, and 72 h without food. For heat and cold treatments, individuals were supplied with fresh *P. armandii* phloem as food, with exposure durations matching those of the starvation assays. Untreated, freshly collected *D. armandi* served as controls for each treatment. Each treatment group comprised 20 individuals, with three biological replicates.

### 4.4. Expression Pattern Analysis (Real-Time-qPCR)

*β-actin* [72] and *CYP4G55* [73] were used as internal reference genes for normalization. Real-time qPCR primers for *DaAKH* and its receptor *DaAKHR* were designed using Primer Premier 5.0 based on the available sequences (Table S6). Primer performance was subsequently validated by assessing amplification efficiency, reproducibility, and product specificity (single amplicon).

### 4.5. RNAi Analysis

#### 4.5.1. Synthesis of Double-Strand RNA (dsRNA)

Gene-specific primers targeting conserved regions of *DaAKH* and *DaAKHR* were designed (Table S7). PCR amplification was performed using the pMD18-T plasmid (TaKaRa, Dalian, China) containing the full-length target gene sequences as templates. The purified PCR products were subsequently used as templates for in vitro synthesis of *DaAKH* and *DaAKHR* dsRNA with the T7 RiboMAX™ Express RNAi System (Promega, Madison, WI, USA). The resulting dsRNA products were stored at −80 °C until use.

#### 4.5.2. RNAi Procedures

Prior to injection, *D. armandi* adults and larvae were anesthetized on ice for 20 min to reduce activity [74]. Injection volumes of dsRNA solution were 0.2 μL for adults and 0.1 μL for larvae [71], delivered using a Hamilton Microliter syringe (Hamilton, Bonaduz, Switzerland). Uninjected insects and insects injected with DEPC-treated water were used as controls. A dsGFP negative control was not included in the main experiments because supplementary validation confirmed that non-homologous dsRNA did not cause sequence-independent gene regulation or immune stress responses under our experimental conditions (Figure S1). The primer sequences for dsGFP are provided in Supplementary Table S7. The uninjected group served to establish the baseline physiological state of the beetles without any external manipulation, while the DEPC-treated water-injected group was used to control for possible stress or physical effects arising from the injection procedure and solvent, thereby ensuring that observed differences could be attributed specifically to the RNAi-mediated gene knockdown rather than the manipulation itself. Beetles were subsequently subjected to starvation, heat, or cold stress. For each treatment, 120 individuals were used (40 larvae, 40 female adults, and 40 male adults); within each stage/sex, 20 individuals were allocated to assess RNAi knockdown efficiency and the remaining 20 to measure post-interference mortality and metabolite contents. Three independent biological replicates were performed for each treatment.

#### 4.5.3. Assessment of RNAi Knockdown Efficiency

Knockdown efficiency was assessed at 72 h post-injection based on prior RNAi studies in Coleopteran insects and our preliminary experiments in *D. armandi* [8,9,17,71], which indicated that dsRNA-mediated suppression of target transcripts typically reaches a stable and maximal level between 48 and 72 h. This time point was also chosen to minimize acute stress responses observed within the first 24–48 h after microinjection, thereby ensuring that metabolic measurements reflect a physiologically stable knockdown state. From the cohorts designated in Section 4.5.2 for knockdown-efficiency assessment (larvae and female and male adults, 20 individuals each), six individuals were collected at 72 h post-injection and stored at −80 °C. Total RNA was extracted and reverse-transcribed into first-strand cDNA, which served as the template for qRT-PCR to quantify RNAi knockdown efficiency, following the procedures described above.

#### 4.5.4. Assessment of Mortality

Mortality was used as the primary phenotypic endpoint to evaluate the effects of RNAi on *D. armandi* survival under the indicated stress conditions. Then, 72 h after dsRNA injection, individuals specified in Section 4.5.2 for mortality assays were held at room temperature for 1 h. The cohorts consisted of larvae, female adults, and male adults. Each stage or sex group contained 20 individuals. Beetles that remained completely immobile were classified as dead [75]. Mortality was then determined for larvae, female adults, and male adults across the treatment and control groups.

#### 4.5.5. Metabolite Quantification

Metabolite assays were performed 72 h after dsRNA injection. The quantified metabolic parameters were considered critical physiological phenotypic markers for assessing the effects of RNAi on the ability of *D. armandi* to adapt to stress conditions. This timing was selected based on preliminary trials in *D. armandi* under starvation, heat, and cold stress. Using the same time point for gene knockdown validation and metabolite profiling ensured that all measurements were taken under a consistent physiological state, thereby improving data comparability. At 72 h after dsRNA injection, surviving larvae, female adults, and male adults from the starvation, temperature stress (high and low), and control groups described in Section 4.5.4 were collected. The levels of TAG, FFA, glycogen, and trehalose were quantified following the manufacturer’s instructions for the corresponding assay kits (Beijing Boxbio Science & Technology Co., Ltd., Beijing, China). Three biological replicates were included for each treatment and control group.

### 4.6. Statistical Analysis

Relative gene expression levels were calculated using the 2^−ΔΔCt^ method. All statistical analyses were performed in SPSS 19.0 (IBM, Chicago, IL, USA). Differences among multiple groups were assessed using one-way analysis of variance (one-way ANOVA), followed by Tukey’s multiple comparisons test (*p* < 0.05). Comparisons between two groups were performed using independent-samples *t*-tests. Figures were generated in GraphPad Prism 6.0 (GraphPad Software, San Diego, CA, USA).

## 5. Conclusions

In summary, we cloned the *DaAKH* gene and its receptor gene, *DaAKHR*, from *D. armandi*. Both genes exhibited differential expression across developmental stages and tissues. Under starvation and temperature stresses, *DaAKH* and *DaAKHR* displayed distinct response patterns. RNAi experiments indicate that *DaAKH* and *DaAKHR* are involved in the regulation of energy metabolism under starvation, heat, and cold conditions. Interference with either gene impaired the beetle’s ability to maintain energy homeostasis under environmental stress.

## Figures and Tables

**Table 1:** *DaAKH* and *DaAKHR* amino acid sequence similarity comparison with other insects.

Genes	Genetic Information of Other Insects			
	Species	Name	Accession No.	Identity ^1^
*DaAKH*	*Dendroctonus ponderosae*	AKH	ENN81145.1	93%
	*Neotermes castaneus*	AKH	AML80825.1	65%
	*Zootermopsis nevadensis*	AKH	AML80834.1	58%
*DaAKHR*	*Dendroctonus ponderosae*	AKHR	XP_048517977.1	97%
	*Anthonomus grandis grandis*	AHKR	XP_050295995.1	80%
	*Euwallacea similis*	AKHR	XP_066249755.1	74%

^1^ As predicted by BLAST in NCBI (www.ncbi.nlm.nih.gov, accessed on 15 February 2024).

**Table 2:** Physicochemical properties of *DaAKH* and *DaAKHR*.

Gene	ORF Size (aa/bp)	MW (kDa)	pI	Signal Peptide Prediction
*DaAKH*	72/219	8.24	5.22	SP 0.9971 other 0.0029
*DaAKHR*	376/1131	43.24	9.33	SP 0.0011 other 0.9989

pI: isoelectric point; MW: molecular weight; ORF: open reading frame; SP: secretory pathway signal peptide.

## Data Availability

The data generated and/or analyzed during the course of this study are available from the corresponding author upon reasonable request.

## Supplementary Materials

The following supporting information can be downloaded at https://www.mdpi.com/article/10.3390/ijms27062724/s1.

## Abbreviations

The following abbreviations are used in this manuscript:

AKH	Adipokinetic hormone
AKHR	Adipokinetic hormone receptor
RNA i	RNA interference
ILPs	Insulin-like peptides
HSPs	Heat shock proteins
AFPs	Antifreeze proteins
GPCR	G protein-coupled receptor
TAG	Triacylglycerols
DAG	Diacylglycerol
FFA	Free fatty acid
APCs	AKH-producing neuroendocrine cells
RACE	Rapid amplification of cDNA ends
NPF	Neuropeptide F
NPFR	Neuropeptide F receptor
sNPF	Short neuropeptide F
sNPFR	Short neuropeptide F receptor
