# Supplementary material for: Functional Analysis of Adipokinetic Hormone and Its Receptor Genes in Regulating Energy Metabolism Under Stress Conditions in Dendroctonus armandi
Source: Int J Mol Sci. 2026 Mar 17;27(6):2724. doi: 10.3390/ijms27062724 (PMC13026165; doi:10.3390/ijms27062724)
Supplement: Supplementary file 1 [file ijms-27-02724-s001.zip › Figure S1.pdf]

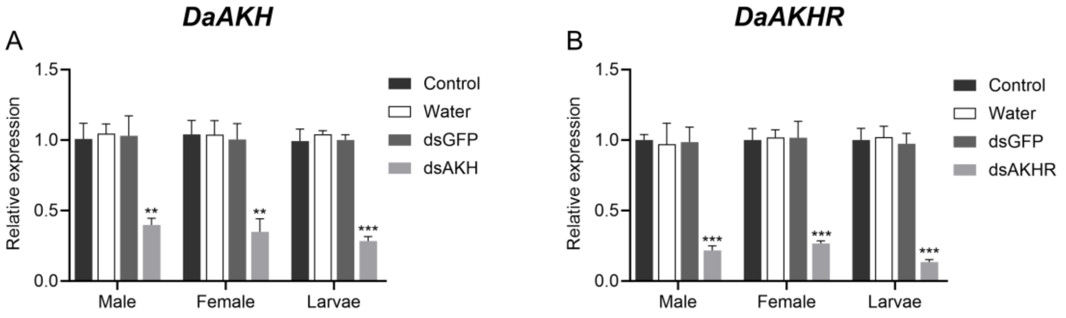

Figure S1. Relative expression levels of *DaAKH* and *DaAKHR* at 72 h post-RNAi. (A) Relative expression levels of *DaAKH* at 72 h post-RNAi treatment in adult males, adult females, and larvae. (B) Relative expression levels of *DaAKHR* at 72 h post-RNAi treatment in adult males, adult females, and larvae. Control: untreated group; Water: DEPC-treated water. The asterisk indicates a significant difference between dsRNA treatment and control groups. (** $p \leq 0.01$, *** $p \leq 0.001$, one-way ANOVA). All values are mean ± SE, n= 3.

The primer sequences for dsGFP are listed in Supplementary Table S7. dsGFP was synthesized using the same protocol as dsAKH/dsAKHR (Section 4.5.1), and injections were performed following Section 4.5.2 (same injection site, volume, and dsRNA concentration as in the main experiments). For the validation assay, four groups were run in parallel: control, DEPC-water injection, dsGFP injection, and dsAKH/dsAKHR injection. Samples were collected at 72 h post-injection under non-stress conditions, and knockdown efficiency was evaluated as described in Section 4.5.3. For each developmental stage/sex, each treatment included three independent biological replicates, with 20 individuals per replicate. Statistical analysis was performed as described in Section 4.6.
