# Supplementary material for: Functional Analysis of Adipokinetic Hormone and Its Receptor Genes in Regulating Energy Metabolism Under Stress Conditions in Dendroctonus armandi
Source: Int J Mol Sci. 2026 Mar 17;27(6):2724. doi: 10.3390/ijms27062724 (PMC13026165; doi:10.3390/ijms27062724)
Supplement: Supplementary file 1 [file ijms-27-02724-s001.zip › Table S3—S7.pdf]

Table S3. Degenerate primers

Genes	Forward primer (5′→3′)	Reverse primer (5′→3′)
DaAKH	GTTTTTGTCTTTTTGGTCTT	GCCTCGTTCTGTATTAATTT
DaAKHR	CTTGCCATTGCTGATTTATT	CCTTCACCGCTTTCCGCTTA

Table S4. Primers for RACE

Genes	Primer sequence (5′→3′)	Primer purpose
DaAKH	TTTTGGTCTTTGTCAGTTTT<br>CAAAGAGTCTGTGGATACCC	3′ RACE
	CCAAAAAGACAAAAACAATC<br>CAGCCATTCTCATTGTCTTC	5′ RACE
DaAKHR	GCTTTCGCACGCCTCTCCAT<br>GCTAAGCAAGGCGAAAACGA	3′ RACE
	AATAAATCAGCAATGGCAAG<br>AAGCACGGCGTAATACCTAT	5′ RACE

Table S5. Full-length primers

Genes	Forward primer (5′→3′)	Reverse primer (5′→3′)
DaAKH	ATTCGACAACTTGCAGTTTTTG	GTTGGAAATCTTCTCACATTCA
DaAKHR	AGAGAAGCAAAGGATTCGCCCG	CTCAAGACTCCTAAGGGAGATT

Table S6. Primers for qRT-PCR

Genes	Forward primer (5′→3′)	Reverse primer (5′→3′)
DaAKH	GGTCTTTGTCAGTTTTTGC	CAGCCATTCTCATTGTCTT
DaAKHR	GCAAAACCAGAAACAAGGG	AGACGAAGAACACAAACAC

Table S7. Primers for RNAi

Genes	Forward primer (5′→3′)	Reverse primer (5′→3′)
dsAKH	TAATACGACTCACTATAGGG<br>TTTTGTCTTTTTGGTCTTTGT	TAATACGACTCACTATAGGG<br>ATTGTCTTCGGACGGGTTGTT
dsAKHR	TAATACGACTCACTATAGGG<br>CATCATCGTGTTTGTGTTCTT	TAATACGACTCACTATAGGG<br>GGGGATAGTCTAGTTTCAGCT
dsGFP	TAATACGACTCACTATAGGG<br>ATGGTGTTCAATGCTTTTCA	TAATACGACTCACTATAGGG<br>CTCTCTTTTCGTTGGGGTCT
